# Supplementary material for: Getting More Out of Biomedical Documents with GATE's Full Lifecycle Open Source Text Analytics
Source: PLoS Comput Biol. 2013 Feb 7;9(2):e1002854. doi: 10.1371/journal.pcbi.1002854 (PMC3567135; doi:10.1371/journal.pcbi.1002854)
Supplement: Dataset S1 — GATE software. Dataset S1 bundled with this paper contains a distribution of GATE (or see http://gate.ac.uk/download/). (TGZ) [file pcbi.1002854.s001.tgz › plos-gate/plugins/Tagger_MetaMap/doc/allclasses-noframe.html]

All Classes


**All Classes**
  

MetaMapPR
  
OutputMode
  
*StringFilter*
  
TaggerMode
  
TextNormalizer
  
TextNormalizer.Java5Normalizer
  
TextNormalizer.Java6Normalizer
